# Transcriptional Profiling in Rat Hair Follicles following Simulated Blast Insult: A New Diagnostic Tool for Traumatic Brain Injury

**DOI:** 10.1371/journal.pone.0104518

**Published:** 2014-08-19

**Authors:** Jing Zhang, Lisa Carnduff, Grant Norman, Tyson Josey, Yushan Wang, Thomas W. Sawyer, Christopher J. Martyniuk, Valerie S. Langlois

**Affiliations:** 1 Chemistry and Chemical Engineering Department, Royal Military College of Canada, Kingston, Ontario, Canada; 2 Defence Research and Development Canada – Suffield, Medicine Hat, Alberta, Canada; 3 University of New Brunswick and Canadian River Institute, Fredericton, New Brunswick, Canada; St. Jude Children's Research Hospital, United States of America

## Abstract

With wide adoption of explosive-dependent weaponry during military activities, Blast-induced neurotrauma (BINT)-induced traumatic brain injury (TBI) has become a significant medical issue. Therefore, a robust and accessible biomarker system is in demand for effective and efficient TBI diagnosis. Such systems will also be beneficial to studies of TBI pathology. Here we propose the mammalian hair follicles as a potential candidate. An Advanced Blast Simulator (ABS) was developed to generate shock waves simulating traumatic conditions on brains of rat model. Microarray analysis was performed in hair follicles to identify the gene expression profiles that are associated with shock waves. Gene set enrichment analysis (GSEA) and sub-network enrichment analysis (SNEA) were used to identify cell processes and molecular signaling cascades affected by simulated bomb blasts. Enrichment analyses indicated that genes with altered expression levels were involved in central nervous system (CNS)/peripheral nervous system (PNS) responses as well as signal transduction including Ca^2+^, K^+^-transportation-dependent signaling, Toll-Like Receptor (TLR) signaling and Mitogen Activated Protein Kinase (MAPK) signaling cascades. Many of the pathways identified as affected by shock waves in the hair follicles have been previously reported to be TBI responsive in other organs such as brain and blood. The results suggest that the hair follicle has some common TBI responsive molecular signatures to other tissues. Moreover, various TBI-associated diseases were identified as preferentially affected using a gene network approach, indicating that the hair follicle may be capable of reflecting comprehensive responses to TBI conditions. Accordingly, the present study demonstrates that the hair follicle is a potentially viable system for rapid and non-invasive TBI diagnosis.

## Introduction

Blast-Induced Neurotrauma (BINT) has become an issue for soldiers since high explosives were introduced to warfare in the mid-19^th^ century. BINT is considered a source of traumatic brain injury, or TBI [1]. TBI describes any brain injury caused by the mechanical impact to the cranium. It has been considered to be a major cause of war casualties due to the wide adoption of blast-orientated modern weaponry reported first in 1916 by British medical director Major Fredrick Mott, based on his post-mortem examinations on two WWI soldiers succumbed to blast injury [2]. In fact, soldiers experiencing damage from explosive devices composed 74% of total war casualties from Operation Enduring Freedom (OEF) and Operation Iraqi Freedom (OIF) [3]. Increased number of war casualties from those recent military activities has placed the matter of TBI under the spotlight of news media and the research community [4]–[7]. Impacts of TBI to the brain can be categorized into either primary or secondary damages. Primary damages are the acute injury consequences to the brain that usually occur immediately after exposure to traumatic conditions (e.g. penetrating brain injury); whereas secondary damages refer to the prolonged effects of post initial trauma, which are usually reflected on a cellular or subcellular level [8]–[9]. Thanks to the advanced modern operational medicine and effective implementation of body armour during combat, the acute consequences (primary damage) caused by explosive-induced TBI has been greatly reduced. That is, the initial exposure to traumatic condition may not accompany immediate or acute damage to the subject. As a result, most battlefield blast related TBIs are considered as mild TBI (mTBI). It was reported that of 266,810 documented TBI cases in US service member, approximately 75% were classified as mTBIs [10]. Therefore, secondary damage of mTBI is particularly vital in this case. The clinical symptoms of blast associated mTBI may include headaches, confusion, short-term memory loss, changes in cognition; changes in personality, vertigo, tinnitus and seizures [4]–[5].

Current TBI/mTBI research has focused on two interconnected streams: investigating molecular pathology and exploring efficient diagnostic targets. A good understanding of the molecular mechanisms behind mTBI symptoms would lead to the discovery of specific biomarkers that can be used for diagnostic purposes [11]. At present, the primary sources for TBI research are blood and brain tissues from either TBI patients or laboratory rodent models [11]–[15]. Although it is crucial for a thorough understanding of TBI (especially the primary injuries) by directly examining the locales of injury, it would be beneficial to utilize an equally robust system that accurately reflects the molecular consequences from TBI exposure and, at the same time, be conveniently translated into a biomarker system. It is also well established that different cells house key information that is both specific to their type as well as communication between them under a given environmental and/or medical condition. Therefore, a system containing multiple cell types would provide a comprehensive molecular signature network under TBI conditions, especially for the secondary injuries. Such a system can be selected based on (a) level of exposure to traumatic conditions that may induce TBI, (b) versatility of molecular responses during or after impact, and (c) accessibility. With the current prevalence of blast-induced TBI in veterans returning from recent conflicts, a non-invasive diagnostic methods or biomarker system that can be used to determine the severity of TBI both efficiently and effectively is in demand.

The mammalian hair follicle system represents a well-coordinated and highly sophisticated process that requires crosstalk between specialized cells, ranging from follicular stem cell populations to fully differentiated cells such as keratinocytes [16]–[19]. Hair morphogenesis and hair cycle progression are governed by underlying cellular signal transduction as well as the subsequent regulation of gene expression. Therefore, the mammalian hair follicle system has the potential to sense environmental stress and produce corresponding physical and/or molecular responses. In fact, it has been reported that the hair follicle is capable of responding to various stressors such as ultrasound and sensitive to the neurohormones, neurotransmitters as well as cytokines whose release is triggered by conventional stress responses [19]–[20]. In addition, hair follicles can be easily obtained by simple hair plucking, which requires no professional skills. Accordingly, the mammalian hair follicle system may be an optimal candidate for TBI research and a potential source for biomarker exploration for diagnosis.

At its core, secondary damage of mTBI is a consequence of altered biological signal transduction and gene expression under traumatic conditions. Stress responsive signal pathways and gene expression subsequently lead to adjustment to metabolism and cellular processes. Studies have shown the involvement of pathways including G Protein-coupled Receptors (GPRs), Toll-Like Receptors (TLRs), Janus Kinase (JAK)/Signal Transducer and Activator of Transcription (STAT) as well as Mitogen-Activated Protein Kinase (MAPK) signaling cascades in TBI responses [21]–[23]. It is also not a surprise that mTBI interrupts calcium and potassium homeostasis, leading to release of the neurotransmitter glutamate, which subsequently initiates cell death cascades [24]–[25]. Other key secondary injuries after the initial trauma include inflammatory responses, synaptic central nervous system (CNS) responses and cell survival/proliferation, among others [26]–[28].

The current study utilizes hair follicles from a blast exposed rat model (*Rattus norvegicus*) to characterize gene expression patterns in the hair follicle. An Advanced Blast Simulator (ABS) was developed and deployed to deliver a single pulse shock wave, and head-only exposures of rats were carried out in a manner that simulated primary blast, and included elements of concussive and whiplash forces. Studies have demonstrated that the amount and quality of hair follicle RNA extract is sufficient for microarray hybridization [29]–[30]. Therefore, a 60K rat microarray was used to identify the TBI responsive gene expression pattern in rat hair follicles following blast exposure. We hypothesized that hair follicle houses the said molecular signatures identified in other TBI diagnostic and model systems and is able to respond similarly to TBI. We provide proof of principle that the hair follicle can be a useful tissue for assessing and monitoring TBI.

## Materials and Methods

### 2.1. Animal experiment

A custom built advanced blast simulator (ABS; approx. 30.5 cm in diameter and 5.79 m in length) located at DRDC (Defence Research and Development Canada), Suffield Research Centre (Medicine Hat, Alberta, Canada) was used for producing simulated blast waves [31]. Four transparent polycarbonate windows ports were equipped on the shock tube to visually observe the response of rat to shock waves. Two high-speed cameras were also used to conduct high speed photography.

Male adult Sprague-Dawley (SD) rats (350–400 g, 9 to 10 week of age, Charles River, Montrean, QC) were used in the current study. The animals were acclimated to the experimental environment for at least one week before use. For exposure, rats were stabilized in plastic sleeves and 3% isoflurane (in oxygen) anaesthetic was conducted for minimum 8 min. The sleeves were then placed in the ABS with the rat head positioned in the test area for shock wave exposure (20, 25 and 30 psi for approximately 4 msec positive duration). Fig. S1 is a representative of a single shockwave at 25 psi. Other detailed information with regard to blast simulation procedure can be found in supplementary materials.

After exposure, rats were closely observed for signs of stress and general health either 1 or 7 days before being sacrificed. At the end of the observation period, rat whisker follicles (n = 4–8) were quickly pulled and placed in RNAlater (Qiagen, Toronto, ON, CA). Samples were then stored at −80°C until further analysis. In conducting this research, the authors adhered to the “Guide to the Care and Use of Experimental Animals” and “The Ethics of Animal Experimentation” published by the Canadian Council on Animal Care. All experimental procedures were approved by the Animal Care Committee at Defence Research and Development Canada (DRDC).

### 2.2. Sample Preparation

RNA was isolated from a pooled sample of 6–8 rat hair follicles using a Qiagen microkit (Qiagen, Toronto, ON, CA). Briefly, follicle samples were homogenized with a sonicator (Ultrasonic Dismembrator-150T, Thermofisher, Ottawa, ON, CA) and a HiBind RNA Spin Column was then used to purify the RNA. Concentrations and integrity of total RNA were determined with a NanoDrop-2000 spectrophotometer and an Agilent Bioanalyzer 2000 [32]. The mean RNA integrity values (± SD) for all samples used in the microarray analysis was 7.32 (±1.09).

### 2.3. Microarray Analysis

A commercially available SurePrint G3 Rat GE 8×60K (G4853A) was purchased from Agilent (Mississauga, ON, CA). Microarray analysis was performed using hair follicles from three sham control rats and five rats that were exposed to simulated blast. Microarray hybridizations were performed according to the Agilent One-Color Microarray-Based Gene Expression Analysis protocol using Cyanine 3 (Cy3) and followed the protocol outlined in Langlois and Martyniuk (2013). Briefly, 200 ng total RNA/sample was used to produce cRNA (Agilent Low RNA Input Fluorescent Amplification Kit). Fragmentation of the cRNA (Agilent, Gene Expression Hybridization Kit) was followed by 17 h hybridization at 60°C. An ozone barrier slide was used to cover rat microarrays before being scanned using an Agilent High Resolution DNA Microarray Scanner. Expression data from .tif images were extracted by the Feature Extraction Software (v10.7.3.1). The quality of microarray data was evaluated by manual inspection of the quality control reports provided from the Agilent software and each microarray was deemed to be of high quality. Raw expression data was imported into JMP Genomics v5.1. Raw intensity data for each microarray was normalized using Quantile normalization and differentially regulated transcripts were identified, as determined using an one-way analysis of variance (ANOVA) followed by a False Discovery Rate (FDR) set at 5%. All raw microarray data for this experiment have been deposited into the NCBI Gene Expression Omnibus (GEO) database (GSE46367).

A subset of genes was selected and subjected to qPCR analysis for validating microarray results. The genes were angiotensin I converting enzyme 2 (Ace2), Tlr2, phosphoprotein enriched in astrocytes 15a (Pea15a), AHNAK nucleoprotein (Ahnak), tp53 regulated inhibitor of apoptosis 1 (Triap1), actin related protein 2/3 complex subunit 4 (Arpc4), Stat5a, spindle and kinetochore associated complex subunit 2 (Ska2), serpin peptidase inhibitor (Sperini), serpin peptidase inhibitor clade A (Serpina1) and hemoglobin beta (Hbb). All the qPCR gene targets were involved in key cellular processes that were reported to be TBI responsive (see the discussion section for details). In addition, elongation factor-1 alpha (Ef1a) used as housekeeping genes for standardization. Detailed procedure is described in supplementary materials. Primer set and optimized qPCR conditions of each gene can be found in Table S1.

### 2.4. Microarray data analysis

Intensities were first filtered in JMP Genomics using the average negative control intensity, the lowest two points on the standard curve (Agilent spike in controls) and the dark intensity signal. Using these metrics, the limit of detection was estimated to be an intensity of 3.0 and all spots measured below this intensity were assigned a value of 3.0. Based upon the quality control report, Feature Extraction, no probe on the rat microarray was saturated. Functional enrichment for gene ontology was performed in JMP Genomics. A *p*-value of 0.05 was the binary cut-off for the Fisher's Exact Test.

Gene set enrichment analysis (GSEA) was conducted using Pathway Studio 9.0 (Ariadne, Rockville, MD, USA) and ResNet 9.0. The number of gene probes mapped in Pathway Studio was 39, 422. For duplicate probes, the probe that showed the lowest *p*-value (i.e. most significant) was used for gene set ranking and the background list for GSEA was the rat genome. Genes were permutated 1,000 times using the Kolmogorov-Smirnov classic approach as an enrichment algorithm. To broaden the analysis, all pathways were expanded to include cell processes and functional classes in target gene seeds. Gene set categories examined for enrichment within the microarray data included the curated Ariadne cell signaling and metabolic pathways as well as Gene Ontology (GO) terms. Sub-network enrichment analysis (SNEA) for proteins and chemicals regulating cell processes was also performed to identify gene networks regulated in hair follicles following blasting (*p*-value was set at *p*<0.05). Additional information on SNEA can be found in Martyniuk and Langlois and Chishti et al. [32]–[33]. Positional GSEA was used to analyze chromosome and chromosomal region enrichment.

## Results

### 3.1. Animal experiments

No obvious signs of injury were exhibited in animals immediately after shock wave exposure. Rats from experimental groups also showed no appreciable difference from sham control animals regarding the time to revive from anaesthetic as well as the overall mobility after revival. Prolonged observation (seven days post exposure) also showed no visible injury in the animals experiencing shock wave or weight change comparing between test and sham control rats. Position and movement of the rat head upon shock wave challenge was analyzed using high-speed cameras and “Tracker” computer software (www.cabrillo.edu/~dbrown/tracker/) (Fig. S2, S3). The kinematic results showed that the head of animals displaced from its initial position according to the strength of the shock wave. The results also suggested that the position of the metal pin used for stabilizing animals greatly influenced the path it took to return to the initial head position. The detailed description of the results upon shock wave exposure can be found in supplementary materials.

### 3.2. Microarray analysis

There were 1,396 gene probes that were differentially expressed at a *p*-value<0.05. There were 646 transcripts that had a ±1.5-fold change (*p*<0.05). Transcripts that showed a 10-fold decrease in the follicles included tyrosine phosphatase-like A domain containing 2 (Ptplad2), arachidonate 12-lipoxygenase epidermal (Alox12e) and coiled-coil domain containing 68 (Ccdc68) while transcripts showing a 10-fold increase included basic helix-loop-helix family member e23 (Bhlhe23), hemoglobin alpha (Hba), hemoglobin adult chain 2 (Hba-a2) and hemoglobin beta (Hbb). The complete list of differentially expressed genes is found in Appendix S1. Appendix S5 is an abbreviation list containing all the genes mentioned throughout the study. A subset of differentially expressed genes was selected from the list to conduct data validation via real-time RT-PCR (qPCR). Microarray data was significantly correlated to qPCR results (R^2^ = 0.66, *p* = 0.004) (Fig. S5). Detailed description of qPCR results can be found in supplementary material.

### 3.3. Gene ontology (GO) term analysis

**Figure 1:**
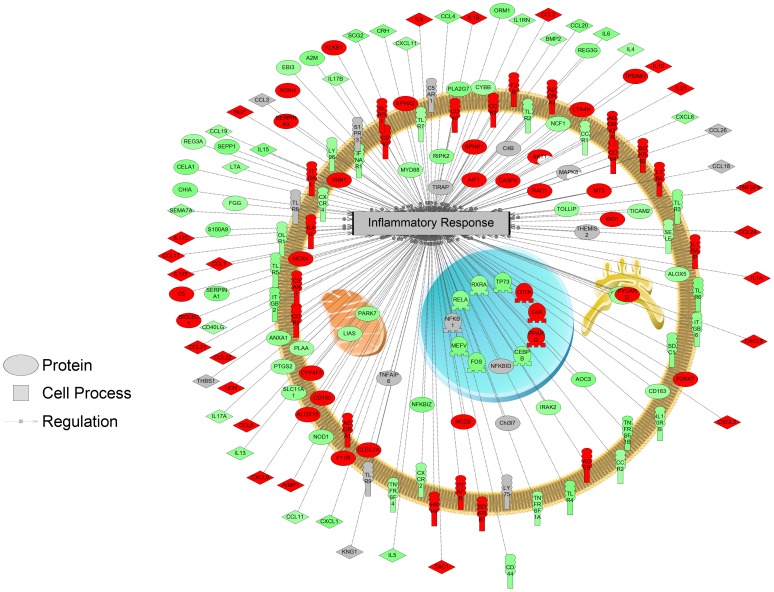
Inflammatory responses enriched by GO term analysis. Red indicates that the gene is increased and green indicates that the gene is decreased in mRNA levels.

Gene ontology functional analysis provided a first glimpse of the biological relevance of the potential TBI responses in mammalian hair follicle. Raw data were analyzed and enriched independently into three main domains: biological processes, molecular function and cellular component (the complete GO term list can be viewed in Appendix S2). For biological processes, 112 GO terms were enriched with *p*≤0.01 upon blast exposure in rat hair follicles. As shown in Table S2A, major themes include cell signal transduction, CNS response (synaptic), stress responsive cell survival and proliferation, and inflammatory response. The TLR signaling pathway represents a major theme under the cell signaling category as multiple related GO terms were enriched. Genes related to 42 GO terms under this category were over-represented with *p*≤0.01 when comparing control rats with the animals after blast exposure (Table S2B). Enriched molecular functions based on the microarray analysis were those support metabolism, signal transduction, hair follicle specific processes, etc. Table S2C shows a selection of enriched GO terms under cellular component (*p*≤0.01). Enriched genes were primarily associated with extracellular spaces (extracellular space, extracellular region, extracellular matrix, proteinaceous extracellular matrix, fibrinogen complex, hemoglobin complex), plasma membrane (plasma membrane, bleb, stored secretory granule), reticulum (sarcoplasmic reticulum, endoplasmic reticulum lumen) and nuclear chromosome. Visual representations of gene networks involved in the inflammatory responses and Ca^2+^ homeostasis can be found in Fig. 1 and Fig. S4, respectively.

### 3.4. Gene set enrichment analysis (GSEA)

**Figure 2:**
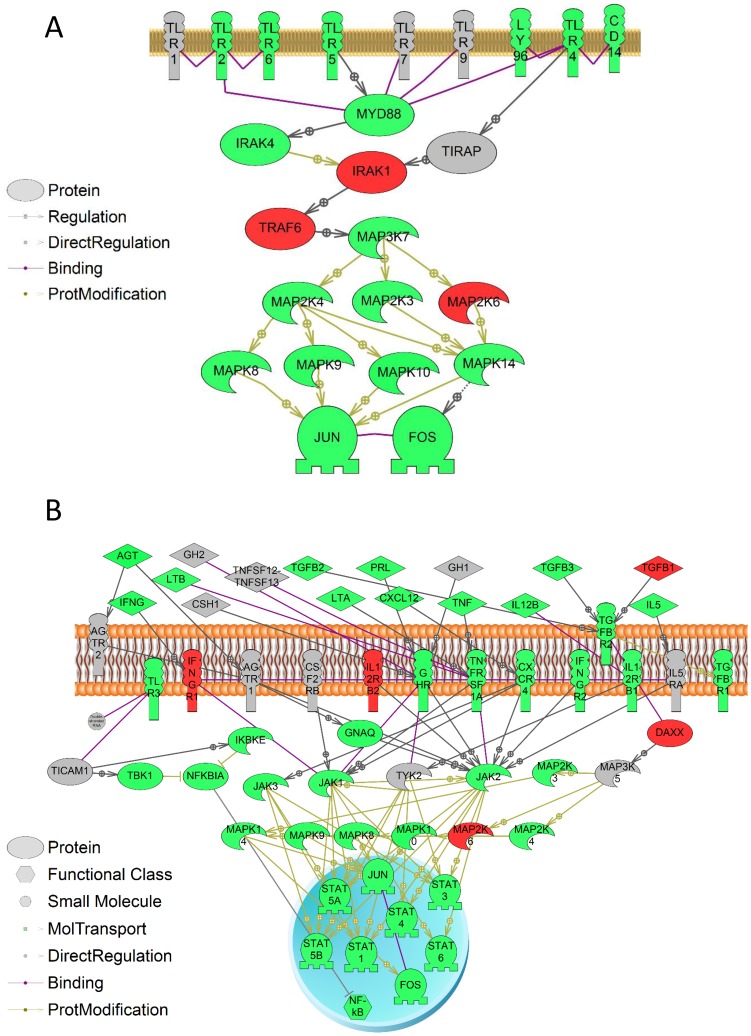
Selected pathways enriched by GSEA analysis. Red indicates that the gene is increased and green indicates that the gene is decreased in mRNA levels. (A) Factors involved in TLR/MAPK pathway; (B) factors involved in JAK/STAT pathway pathways for GH, prolactin, and interleukin signaling.

Following GSEA, a total of 21 pathways were enriched with statistical significance (*p*<0.05) in hair follicles in rats upon blast simulation. Pathways were further grouped into three categories: receptor signaling (five pathways), cell signaling (seven pathways) and metabolism (nine pathways) (Table 1).Our results suggest that TLR/Activator protein 1 (AP1) and Growth hormone receptor (GHR)/STAT pathways were the two receptor signaling pathways that exhibited the highest statistical significance (*p*≤0.01) compared to other differentially enriched pathways (*p*<0.05), reasoning the responses of these two pathway are more likely to happen. Genes connected to the TLR pathway (Tlr2, Tlr4, Tlr5, and Tlr6) showed a decrease in steady state mRNA levels in the rat hair follicles (Fig. 2A). In addition, AP1 was also reported to be responsive to transforming growth factor beta receptor (TGFBR) upon shockwave exposure (Table 1). Beside the GHR, other receptors including C-X-C chemokine receptor type 4 (CXCR4), interferon-gamma receptor (IFNGR) were connected to STAT signaling (Fig. 2B). To be more stringent, we filtered genes based on intensity that were below the detection limit of the microarray. Cell Signaling pathway enrichment contained 4 processes with *p*≤0.01. The results also suggested that there was an enrichment of genes involved in the guanylate cyclase pathway as well as Adherens Junction Regulation (*p*<0.05) (Table 1). Under the metabolism category, there were 5 pathways with *p*≤0.01

**Table 1:** Pathways enriched from GSEA analysis in rat hair follicles upon blast exposure.

	Expanded # of Entities	# of Measured Entities	Median Change	p-value
**Cell signaling pathways**				
Melanogenesis	694	637	1.01	<0.001
Skeletal Myogenesis Control	589	546	1.01	0.00977
Gap Junction Regulation	661	611	1.02	0.01045
Apoptosis Regulation	624	551	−1.04	0.01091
Guanylate Cyclase Pathway	1219	1093	−1.01	0.01567
Adherens Junction Regulation	696	436	1.00	0.02143
Gonadotrope Cell Activation	728	682	1.01	0.04902
**Receptor signaling pathways**				
TLR→AP-1 signaling	30	23	−1.20	0.01339
GHR→STAT signaling	12	9	−1.22	0.01415
TGFBR→AP-1 signaling	16	15	−1.32	0.02871
CXCR4→STAT signaling	9	9	−1.22	0.04386
IFNGR→STAT signaling	8	8	−1.21	0.04902
**Metabolic pathways**				
Arachidonic acid metabolism	251	126	−1.05	<0.001
Metabolism of estrogens and androgens	201	94	−1.03	<0.001
Vitamin B6 (pyridoxine) metabolism	25	8	1.27	<0.001
Heme oxidation	64	22	1.03	<0.001
Irinotecan metabolism	52	27	1.16	<0.001
Tryptophan metabolism	326	150	−1.01	0.0249
Alanine metabolism	48	14	−1.06	0.03153
omega-6-fatty acid metabolism	207	112	−1.04	0.03659
omega-3-fatty acid metabolism	221	125	−1.02	0.0463

### 3.5. Positional GSEA

Chromosomal positional GSEA was carried out to identify specific chromosomal regions that were significantly enriched in the rat genome upon blast exposure in rat hair follicles. In response to blast, differentially expressed genes (*p*<0.05) were located preferentially on a couple of rat chromosomes (Table 2). Chromosomes 1, 6 to 10, 12, 13, 15 and 17 exhibited multiple enriched regions responsive to blast exposure, among which chromosomes 1 (1p13, 1q32, 1q43, 1p12), 7 (7q22, 7q11, 7q13) and 13 (7q22, 7q11, 7q13) encompassed the chromosomal regions that contained genes most affected by blast simulation.

**Table 2:** Chromosome enrichment identified regions of the rat genome that contain groups of genes that preferentially increased or decreased in mRNA levels in the hair follicle.

Chromosome	Position	Total Entities	Measured Entities	Median change	p-value
1	1p13	78	29	−1.10	<0.001
	1q32	329	123	1.07	<0.001
	1q43	369	199	1.10	<0.001
	1p12	103	43	−1.08	0.0218978
2	2q41	70	38	−1.00	0.0306346
3	3	689	43	−1.00	0.0360531
5	5q24	134	61	−1.21	<0.001
6	6q33	118	16	−1.25	0.00183824
	6q32	236	121	1.05	0.0310966
7	7q22	216	129	−1.05	0.00165837
	7q11	493	189	1.08	0.0270936
	7q13	184	89	−1.05	0.0290909
8	8q23	50	31	−1.17	0.003861
	8q32	313	212	1.05	0.00775194
9	9q37	47	20	−1.15	0.00601202
	9q38	31	18	−1.10	0.0215517
10	10q32.3	144	104	1.15	0.0112108
	10q25	57	40	1.06	0.0263158
11	11q12	109	41	−1.12	0.0419708
12	12p12	99	39	1.02	0.0262238
	12q12	163	81	1.11	0.0484581
13	13p13	55	19	−1.41	<0.001
	13q23	38	23	−1.16	0.0108303
	13q22	85	50	−1.04	0.0441989
15	15q22	23	9	−1.18	0.0258449
	15p12	175	94	−1.03	0.037702
16	16p16	121	79	−1.08	0.0213523
17	17p11	73	34	−1.06	0.0346821
	17q11	81	24	−1.01	0.0395683
X	Xq21	148	66	1.05	0.0386364

**Figure 3:**
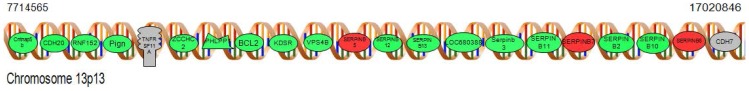
Rat chromosome 13p13 is a significantly enriched chromosomal position. The region has an overall decrease of −1.39-fold. Genes are shown in their position relative to the other genes from position 7,714,565 to 17,020,846. Red indicates the gene in increased in mRNA abundance in the hair follicle while green indicates that the gene is decreased in mRNA abundance.

A number of serine protease inhibitor genes (e.g., Serpinb3, -5, - 11 and -12) on the chromosome 13 position 13p13 were preferentially down-regulated (∼40%) following blast (Fig. 3). Six members of this gene family showed a decrease in expression while three others were increased in mRNA abundance in rat hair follicle. Other genes within this position with decreased transcript levels included B-cell CLL/lymphoma 2 (Bcl2), vacuolar protein sorting 4 homolog B (Vps4b), and phosphatidylinositol glycan anchor biosynthesis (Piga), and phosphatidylinositol-glycan biosynthesis class n (Pign). Interestingly, a region on the X-chromosome was identified as enriched and included gastrin-releasing peptide receptor (Grpr), Gpr 64, -143 and -173.

GSEA was conducted on the genes that were differentially enriched on chromosome 1, 7 and 13, in order to explore the functional significance of those chromosomes in rat hair follicle upon blast exposure. Considering the number of pathways enriched on those chromosomes, it is beyond the scope of the current paper to discuss all the processes. Hence, we focus on the pathways that were also enriched in microarray GSEA analysis described in section 3.4. The complete GSEA results for all three chromosomes can be found in Appendix S3. For the receptor signaling category, our GSEA results on positional enrichment data suggest that genes associated with TLR signaling were enriched on chromosome 1 and 7 while those related to STAT pathways were found on all three chromosomes. Genes involved in TGFBR/AP1 signaling were enriched on chromosome 1. Similar to TLR pathway, GHR signaling was also a represented enriched group on chromosomes 1 and 7. The categories of cell signaling and metabolic pathway also showed overlap between microarray GSEA and GSEA on chromosome 1, 7 and 13. These data support those data that were collected for the microarray GSEA and localizes groups of genes with specific functions to chromosomal regions.

### 3.6. Sub-network enrichment analysis (SNEA)

**Figure 4:**
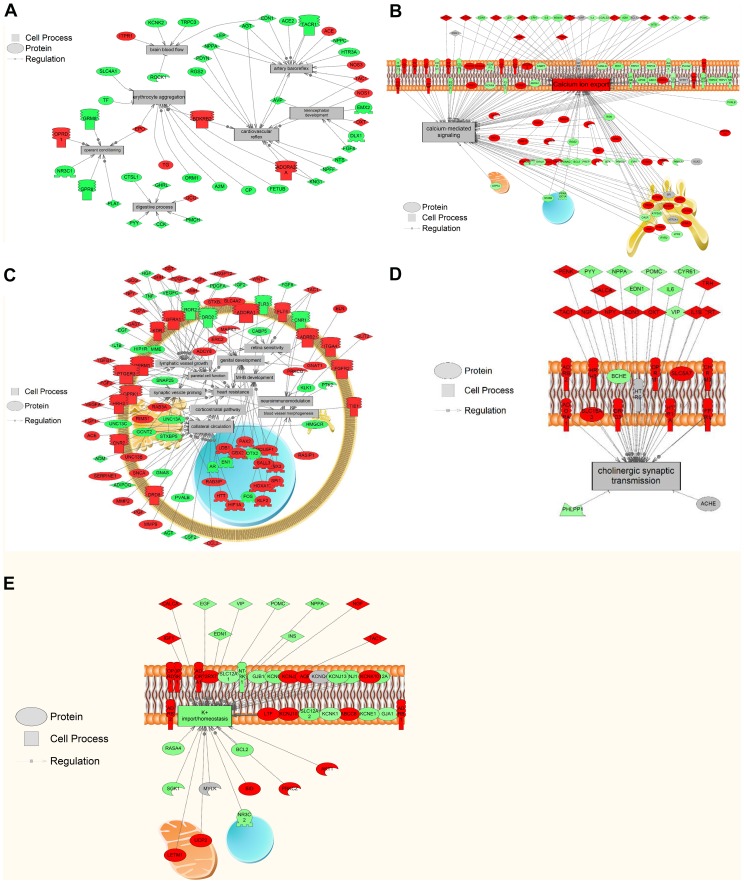
Selected pathways enriched by SNEA analysis. Red indicates that the gene is increased and green indicates that the gene is decreased in mRNA levels. (A) Pathways featuring 45% decrease of genes in transcript level; (B) factors involved in Ca^2+^ export and Ca^2+^-mediated signaling; (C) pathways featuring 35% increase of genes in transcript levels with subcellular distribution; (D) factors involved in cholinergic synaptic transmission with subcellular distribution; (E) factors involved in potassium import/homeostasis with subcellular distribution.

SNEA was conducted to build blast responsive sub-networks in rat hair follicle based on regulatory networks inferred from the literature. Potential upstream regulators, cell processes and related diseases were analyzed. Our results showed a total of 147 potential cell processes were responsive to blast exposure in rat hair follicles with *p*≤0.01 (the complete process list is provided in Appendix S4A). Here we focus on the processes that are closely associated with pathways enriched in GO term and GSEA analysis. Table 3 lists the candidates grouped to that structure provided for the GO term analysis. Similar to GO terms and GSEA analysis, some of the major themes affected by shock wave blasting were signal transduction, metabolism, cell survival/proliferation, brain injury responsive cellular events as well as hair follicle specific responses. There was excellent coverage of many of the annotated gene networks in the rat (>90%) (Appendix S4A). Networks related to cell signaling were, in general, overwhelmingly decreased (2–13%). Genes with up to 45% decrease in transcript levels are shown in Fig. 4A. However, up-regulations were observed for the genes connected to Ca^2+^ export and Ca^2+^-dependent signal transduction (Fig. 4B). In addition, a few of the processes showed potential positive responses reflected by up-regulations on transcript levels of the related genes (Fig. 4C). It is worth noting that a major process identified in the SNEA analysis was synaptic CNS (Table 3). For example, networks related to dopaminergic and cholinergic pathways, neurotransmitter secretion and uptake, and transcripts involved in nervous system physiology and action potential generation were affected in rat hair follicles. Fig. 4D shows differentially expressed genes connected to cholinergic synaptic transmission.

**Table 3:** Pathways and biological processes enriched in SNEA analysis in rat hair follicles upon blast exposure.

	Gene Set Seed	Total # of Neighbors	# of Measured Neighbors	Median change	p-value
**Signal transduction**	Complement activation	143	127	−1.08	5.77E-07
	Regulation of action potential	184	182	−1.03	1.35E-05
	Membrane polarization	117	113	−1.07	2.17E-05
	Chloride ion homeostasis	100	99	−1.13	0.00021
	Membrane depolarization	631	609	−1.04	0.00065
	Generation of action potential	75	75	1.04	0.00073
	Calcium-mediated signaling	155	149	−1.01	0.00129
	Action potential duration	94	92	1.06	0.00173
	Membrane hyperpolarization	285	283	−1.01	0.00203
	Complement activation, classical pathway	59	50	−1.03	0.0028
	K+ import/homeostasis	67	67	−1.09	0.00338
	Cl- transport	254	247	−1.04	0.00359
	Sarcoplasmic reticulum calcium release	45	41	−1.04	0.00481
	Ion transport	206	204	−1.12	0.00877
	Intracellular signaling cascade	128	126	−1.04	0.00918
	Inflammatory response	1087	1001	−1.05	0.01298
**Cell survival/Proliferation**	Quiescence	261	249	−1.13	0.01233
**on CNS responses**	Ganglion neurotransmission	21	21	−1.28	0.00014
	Transmission of nerve impulse	592	580	−1.03	0.0017
	Nervous system physiology	49	49	1.06	0.02443
	Neuromuscular synaptic transmission	41	39	1.12	0.00243
	Neuronal activity	263	259	−1.01	0.0009
	Neurotransmitter secretion	258	250	1.02	0.01608
	Neurotransmitter uptake	163	160	−1.08	6.72E-05
	Synaptic transmission	506	491	−1.03	0.00297
	Synaptogenesis	202	196	−1.07	0.00397
	Cholinergic synaptic transmission	55	53	−1.13	0.01073
				−1.04	
**Other TBI-responses**	Tissue remodeling	230	216	1.04	0.00046
	Vascularization	1545	1395	−1.01	0.00687
	Vasodilation	396	385	1.06	0.0002
	Adipocyte differentiation	394	367	−1.01	0.00854
	Arteriogenesis	103	101	−1.03	0.0005
	Blood circulation	390	381	−1.09	0.00063
	Blood vessel permeability	300	291	−1.04	0.00015
	Hemato-encephalic barrier	215	212	−1.04	0.00203

Other themes that were identified using SNEA included reproduction, behavioural regulations, hormonal control (Table 3) and potassium homeostasis (Fig. 4E). Reproductive gene networks such as copulation, sperm entry, estrus, ovary function, and proestrus were also affected and the majority of sub-networks related to reproduction were decreased in hair follicles. Behavioral-related gene networks were all decreased in median fold change. In terms of hormonal control, vasopressin and adrenocortical secretion were down-regulated.

SNEA analysis is also capable of mapping differentially expressed genes that are related to diseases. A total of 260 diseases were identified as enriched in the hair follicles from blast exposed rats (*p*<0.05, the complete list can be viewed in Appendix S4B), of which 94 conditions were significant at *p*≤0.01. Major themes include ion homeostasis (e.g., hyponatremia and hypokalemia), heart disease (e.g., arteriolopathy, ventricular fibrillation, aortic valve stenosis, etc.), blood circulation disorders (e.g., artery stenosis, hemodynaic abnormalities, etc.), and inflammation (e.g., granuloma and inflammatory lesions), among others.

## Discussion

To test the hypothesis that hair follicle gene expression is sensitive to blast exposure, we analyzed hair follicles of whiskers harvested from rats exposed to simulated blast in a shock tube specially designed to produce single pulse shock waves. Although this simulator produced shock waves that simulated all the key characteristics of classical free-field blast-wave flow conditions, including the negative phase and secondary shock, the head constraint utilized introduced confounding variables [31]. Kinematic analysis using high speed photography showed that significant concussive and whiplash forces, in addition to the primary overpressure insult, were also produced during the simulated blast exposures. In order to explore the possibility of hair follicle used as a research and diagnostic system for TBI, the current study examines responses of the well-studied brain trauma responsive biological processes such as nervous system response, ion exchange – and GPR – dependent signaling cascades, inflammatory response and apoptosis regulations. High-throughput gene expression profiling presents a robust way of exploring molecular mechanisms behind TBI responses in mammalian hair follicle. The consistent advancement in bioinformatics throughout years has enabled various enrichment algorithms that can be used to analyze the complexity of signal transduction and the related downstream events through multiple types of interaction networks [34]–[38]. Thus the current study makes use of the microarray-enrichment analysis approach to depict the potential similarity of the aforementioned molecular signatures between hair follicle and other established systems including brain and blood.

First of all, the enrichment analyses suggested that synaptic CNS response as a major theme enriched in the rat hair follicles following blast exposure: related GO terms under include regulation of synaptic transmission and synaptic transmission. In fact, synaptic homeostasis and transport are tightly associated to TBI [27]. In particular, the cholinergic neurotransmission impairment was reported in rat models experiencing TBI [39]. It has been revealed that CNS response is a main theme upon TBI [26]–[28]. The SNEA analysis revealed even more synaptic related cellular events, including neurotransmitter uptake, ganglion neurotransmission, neuromuscular synaptic transmission, synaptic transmission, cholinergic synaptic transmission, synaptogenesis, neuronal activity, transmission of nerve impulse, strongly indicating that synaptic homeostasis-related nervous system responses play critical roles in rat hair follicles upon blast. Our results further suggest the genes encoding receptors involved in cholinergic neurotransmission were up-regulated in their transcript levels, including histamine h3 receptor (Hrh3), cholinergic receptor, muscarinic 3 (Chrmm3), opioid receptor mu 1 (Oprm1), solute carrier family 18 member 3 (Slc18a3), adenosine a1 receptor (Adora1), 5-hydroxytryptamine (serotonin) receptor 1a (Htr1a), adrenoceptor beta 2 (adrb2), dopamine receptor d3 (Drd3) and formyl peptide receptor 1(Fpr1). Given its critical roles in nervous system, changes to cholinergic neurotransmission machinery can lead to responses in both CNS and PNS [40]. In addition, TBI responsive synaptic transmission was proposed to be glutamate-related in mammalian brain [41]. In the current study, such regulation was supported by the over-representation of the differentially expressed genes associated to glutamate metabolism. The microarray results suggested regulation of genes encoding related receptors, including glutamate receptor 4 (Gria4) and glutamate receptor ionotropic n-methyl d-aspartate 2a (Grin2a). The GO term domain cellular component confirmed the potential ligand-receptor interactions with enriched subcellular locales of extracellular environment and plasma membrane that house most of the related protein products.

Various types of signal transduction responses were also identified, including ion exchange – and GPR-dependent pathways. Firstly, our microarray and enrichment analyses suggest changes in expression profiles of genes encoding Ca^2+^, K^+^ and Cl^−^ cellular transport and localization. It has been established that the release of excitotoxic amino acids upon TBI triggers alteration in Ca^2+^ transport across cellular membranes as a major secondary injury that is usually associated with cellular damage [42]–[45]. The microarray results suggest that expression levels of genes involved in stimulating Ca^2+^ transport were significantly up-regulated, including Platelet-derived growth factor receptor beta polypeptide (PDGFRB) and Transient receptor potential cation channel subfamily v member 2 (TRPV2) [46]–[47]. SNEA analysis indicated that blast exposed rat hair follicle triggered regulation of genes responsible for sarcoplasmic reticulum calcium release, confirmed by the enriched intracellular locale sarcoplasmic reticulum by GO term analysis. A sustained influx of Ca^2+^ into cells and compartments such as mitochondria and nucleus can initiate apoptotic signaling cascades as it leads a disruption in metabolic processes [48]. Genes involved in K^+^ transport were also enriched by GO term (positive regulation of potassium ion transport, potassium channel inhibitor activity) and SNEA (potassium ion import/homeostasis) analyses. Such regulation was also proposed by Reinert et al. as another ion transmission related secondary injury following severe primary injuries of TBI [24]. It was previously reported that the transmembrane events of chloride ion was regulated by the corresponding adaptations of proteins responsible for capillary permeability upon TBI [49], which was also enriched by SNEA.

Receptor signaling pathway was another enriched signal transduction theme upon blast simulation by the enrichment analyses, as indicated by the GO term receptor clustering. Such pathways include GPR-dependent signaling (GO term enrichment) and its major regulator of the guanylate cyclase pathway (GSEA) [21]. It is well documented that GPRs are able to trigger signal transduction through key pathways and are crucial for mediating cellular responses to medical disorders [50]–[51]. Specifically, it was reported that GPRs were regulated in response to TBI in rat brain [52]. One example of GPR-mediated signal transduction is MAPK pathway [53], which was also enriched by GO term analysis. MAPK signaling cascades consist of three main kinase pathways: extracellular signal regulated kinase (ERK), c-Jun N-terminal kinase (JNK) and p38 MAPK, which are sensitive to various environmental change and stress conditions [54]. It was proposed that all three MAPK pathways were TBI sensitive [23]. GPR also facilitates JAK/STAT signaling, which is also TBI-responsive [12], [55]. GPR-based activation of JAK/STAT pathway requires Rho GTPase activity [56]. Both GO term and GSEA analyses showed that genes involved in JAK/STAT pathway and regulations on Rho GTPase were over-represented, indicating the Rho GTPase-dependent GPR/JAK/STAT a potential in rat hair follicles upon blast simulation.

TLR pathway is another signaling pathway affected at transcriptomic level in hair follicles following blast (GO term and GSEA analyses). It was previously demonstrated that TLR pathways were responsive to TBI in the brain of the mice and proposed as a biomarker for stroke in blood [22], [57]. Many of the TLRs mapped had a decrease in transcript level following blast, namely: Tlr2, Tlr4, Tlr5 and Tlr6. TLRs are signaling molecules that assist in the regulation of the immune response to tissue damage. The qPCR analysis also showed consistent decrease in transcript level of Tlr2, further confirming the results observed in microarray analysis. TLRs also function as upstream receptors to MAPK cascades [58]. Our results suggest potential TLR-dependent inhibition of MAPK signal transduction associated with down-regulated transcript levels of Tlrs and genes involved in MAPK pathways. Therefore, it appears that rat hair follicles are capable of responding to TBI conditions similar to mammalian brain in terms of regulation on TLR pathways.

As mentioned above, some of the enriched signaling pathways upon blast exposure are directly linked to inflammatory responses, which is another well-known TBI response [26]. The enriched JAK/STAT and TLR/NFkB pathways are major upstream signaling cascades that are able to trigger inflammatory response [12], [59]. The JAK/STAT-mediated inflammatory responses are cytokines-dependent (e.g. interleukins (IL) [60]. Our GO term analysis revealed multiple biological processes and molecular functions enriched towards regulations on IL, indicating potential connections between JAK/STAT/IL-dependent inflammatory responses. The GSEA analysis also suggested additional receptor – STAT interactions that could lead to JAK/STAT-dependent inflammation, such as GHR and IFNGR [61]–[62]. GHR interacts with JAK/STAT signaling through AP1/FUN/FOS pathway [63]–[64]. The microarray results suggested decreased transcript levels of Ifngr2, Jak2 and all the downstream Stat genes, suggesting that the IFNGR2/JAK/STAT pathway may be inhibited in hair follicle upon shock wave exposure. Our qPCR analysis also suggested a decrease of Stat5a transcript level, consistent with the result from microarray. In terms of TLR/NFkB, the results from blast exposed rat hair follicles showed decrease in transcript levels of the related genes. Since TLR/NFkB pathway stimulates immune response, while inhibiting inflammation [65], the results suggested a potential pro-inflammation regulation. Along with the observed decrease of Tlr4 transcript level, the GO term results also showed a decrease in the adaptor molecule myeloid differentiation factor 88 (MYD88). TLR4 is able to exacerbate cell damage in the brain and trigger inflammatory responses following trauma [65]. Activation of TLR4 stimulates NFkB, which in turn affects genes that encode pro-inflammatory molecules. MYD88 is part of the TLR2-MyD88-NFkB pathway that is related to the release of IL-1β, a key mediator in the inflammatory response.

As a result of pro-inflammation signal transduction, multiple inflammatory responses were reported following TBI exposure, including elevation of intracranial pressure, accumulation of polymorphonuclear leukocytes as well as increased proliferation of Natural Killer (NK) cells [66]. Genes involved in such processes were preferentially regulated in the rat hair follicles, as demonstrated in GO term analysis. As shown from the pathway analyses, genes involved in inflammatory response with altered expression profile were significantly enriched. Therefore, shock wave simulated blast exposure led to a full spectrum of inflammatory responses ranging from signal transduction to specific cellular processes, suggesting rat hair follicle is capable of reflecting the complete molecular complexity of brain trauma-induced inflammation.

Furthermore, genes involved in defense response, cellular defense response, response to mechanical stimulus and protein refolding upon were enriched in GO term analysis upon blast exposure. Biological processes related to cell survival and proliferation secondary injuries that are commonly associated with the initial trauma to the brain [28]. Therefore, it is not a surprise that multiple programmed cell death related GO terms and GSEA pathways were enriched. It is worth noting that glutamate metabolism also plays a critical role in neuronal injury-related cell death via gap junction regulation [67]. Consistent with the over-represented genes involved in glutamate metabolism in GO term analysis, gap junction regulation was enriched in GSEA. TBI-associated cell death occurs in both and secondary injuries over prolonged post-traumatic periods [28], [68]. Apoptosis is controlled by both pro- and anti-apoptotic factors responsive to environmental and/or cellular conditions [69]. The anti-apoptotic B-cell CLL/lymphoma 2 (Bcl2) is a critical factor in regulating apoptosis. Altered expression of Bcl-2 gene may lead to an imbalance in the homeostatic equilibrium between cell survival and death [70]. Our microarray data showed that the transcript levels of Bcl2 were significantly down-regulated, suggesting potential apoptosis events in hair follicles upon blast. Studies have shown regulated Bcl2 expressions following brain trauma [71]–[72]. Furthermore, the previously discussed signaling pathways are also involved in cell survival. For example, AP1 transcription factor has also been shown to regulate a host of target genes that are involved in programmed cell death following brain ischemia [73]–[74].

SNEA disease network analysis also suggests that hair follicle is able to reflect comprehensive TBI consequences beyond direct responses. The results suggest that the rat hair follicle system is able to reflect potential changes in blood pressure and related cardiovascular responses upon blast exposure. Cardiovascular homeostasis is a major theme enriched in rat hair follicle and essential to the health of both body and brain. Following moderate to severe TBI, this equilibrium is often disrupted and can result in the loss of blood pressure autoregulation and attenuation of the normal bradycardic response to injury [75]. The gene sub-networks showed significant trends in pathways related to brain blood flow, artery baroreflex and cardiovascular reflex. Many of these pathways showed significantly lower gene expression levels than control (up to a 45% reduction). Magnetic resonance imaging (MRI) assessments of cerebral blood flow performed following experimental TBI in mice have shown that hemorrhagic shock and systemic hypotension resulting from blood loss exacerbate cell death in the hippocampus [76]. Moreover, internal blood surges induced by blast over-pressure have been hypothesized to induce increases in cerebral perfusion pressure and micro-tears in both cerebral blood vessels and blood-brain-barrier (BBB) [77]. The transfer of kinetic energy from the blast causes a “rippling effect” that oscillates blood vessels to the brain causing morphological alterations [7]. These cerebrovascular insults result in BBB breakdown as seen in several animal studies [13], [77]–[78] and may be the reason for the up-regulation in the SNEA of blood vessel morphogenesis pathway seen in our results.

Additionally, positional enrichment analysis suggested that chromosome 1, 7, 13 and corresponding regions on them were preferentially affected by blast simulation. Rat chromosome 1 (the largest rat chromosome) houses the highest number of genes that are responsive to neurological disorders [79]. Chromosome 1 was also considered related to cardiovascular disorders [80]. Previous study has demonstrated a locus on chromosome 7 is involved in blood pressure disorders while chromosome 13 is linked to hypertension [81]–[83], consistent with the SNEA disease enrichment. In addition, a region on the X-chromosome (Xq21) was preferentially responsive to blast simulation, suggesting sex differences in the response to shockwave exposure may exist at the level of the transcriptome.

Accordingly, the current study demonstrated that gene expression was responsive to blast exposure in rat hair follicles. Multiple gene set enrichment analyses suggested that several types of cell signal transduction cascades, cell survival and nervous system responses. The results suggested that, on a molecular level, rat hair follicle responds to traumatic conditions similarly to brain and blood of both human patients and lab rodent models (e.g., name with pathways are the same). In addition, transcriptomics responses in the rat hair follicle corresponded to brain trauma related medical disorders as well as potential TBI-responsive chromosome positional enrichment. These data indicate that hair follicle may be a robust system with similar TBI molecular signatures that have been characterized in other systems. Furthermore, hair follicles can be easily obtained via simple plucking without professional skills. These features make hair follicle an optimal biomarker system for TBI in the context of military activities as well as other traumatic conditions that could lead to brain injury. In conclusion, the present study demonstrated for the first time that mammalian hair follicle is a potentially optimal system for both TBI pathology study and biomarker discovery.

## Supporting Information

Figure S1 **Representative of a single shock wave.** This shock wave is produced with a 25 psi target pressure.
(TIFF)

Figure S2 **Displacement of rat nose in XY plane.** Origin is initial resting position of nose. Origin is initial resting position of nose; the path of movement was tracked using high speed video and tracking the tip of the nose.
(TIFF)

Figure S3 **Radial distance displacement of rat nose from origin.** Origin is initial resting position of nose.
(TIFF)

Figure S4 **Factors involved in Ca^2+^ homeostasis; red indicates that the gene is increased in transcript level.**
(TIFF)

Figure S5 (A) Linear regression analysis showing correlation between microarray and qPCR; fold change to control was plotted for both microarray and qPCR. (B) Comparison of transcript level change in rat hair follicle upon shock wave exposure between microarray and qPCR.
(TIF)

Table S1 **Primers and their corresponding conditions for qPCR analysis.**
(DOCX)

Table S2 Table S2A: Enriched GO terms in rat hair follicles after blast exposure under the domain of Biological Processes. Table S2B: Enriched GO terms in rat hair follicles after blast exposure under the domain of Molecular Functions. Table S2C: Enriched GO terms in rat hair follicles after blast exposure under the domain of Cellular Components.
(DOCX)

Appendix S1 **Gene expression data for the rat transcriptome.**
(XLS)

Appendix S2 Appendix S2A: GO term enrichment, Biological Processes domain. Appendix S2B: GO term enrichment, Molecular Functions domain. Appendix S2C: GO term enrichment, Cellular Components domain.
(XLSX)

Appendix S3 Appendix S3A: Chromosome 1 GESA. Appendix S3B: Chromosome 7 GSEA. Appendix S3C: Chromorsome 13 GSEA.
(XLSX)

Appendix S4 Appendix S4A: SENA analysis. Appendix S4B: SNEA diease analysis.
(XLSX)

Appendix S5 **Abbreviations.**
(XLSX)
