# Supplementary figures and images for: Transcriptional Profiling in Rat Hair Follicles following Simulated Blast Insult: A New Diagnostic Tool for Traumatic Brain Injury
Source: PLoS One. 2014 Aug 19;9(8):e104518. doi: 10.1371/journal.pone.0104518 (PMC4138085; doi:10.1371/journal.pone.0104518)

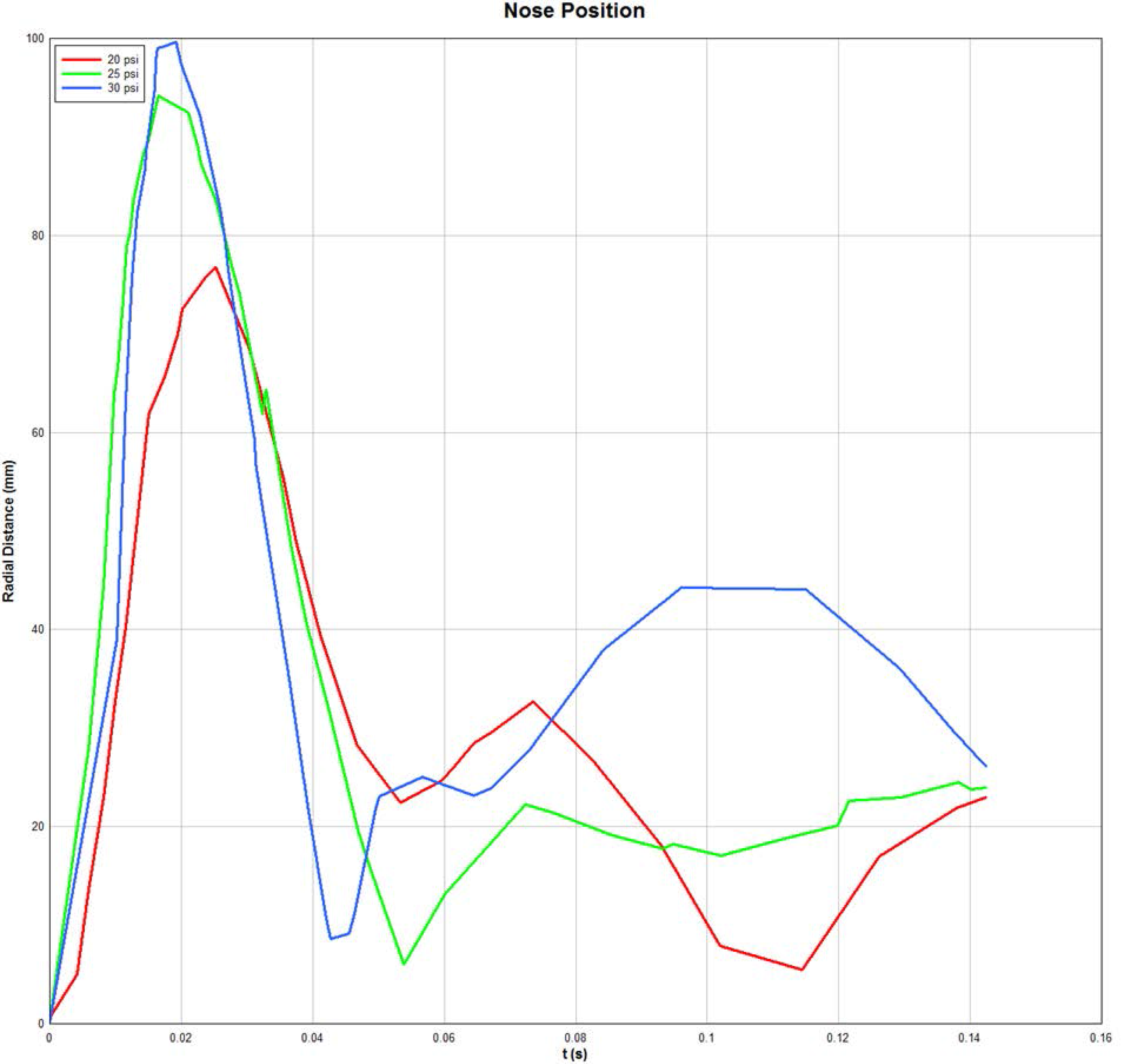

Supplement: Figure S3 — Radial distance displacement of rat nose from origin. Origin is initial resting position of nose. (TIFF) [file pone.0104518.s003.tiff]

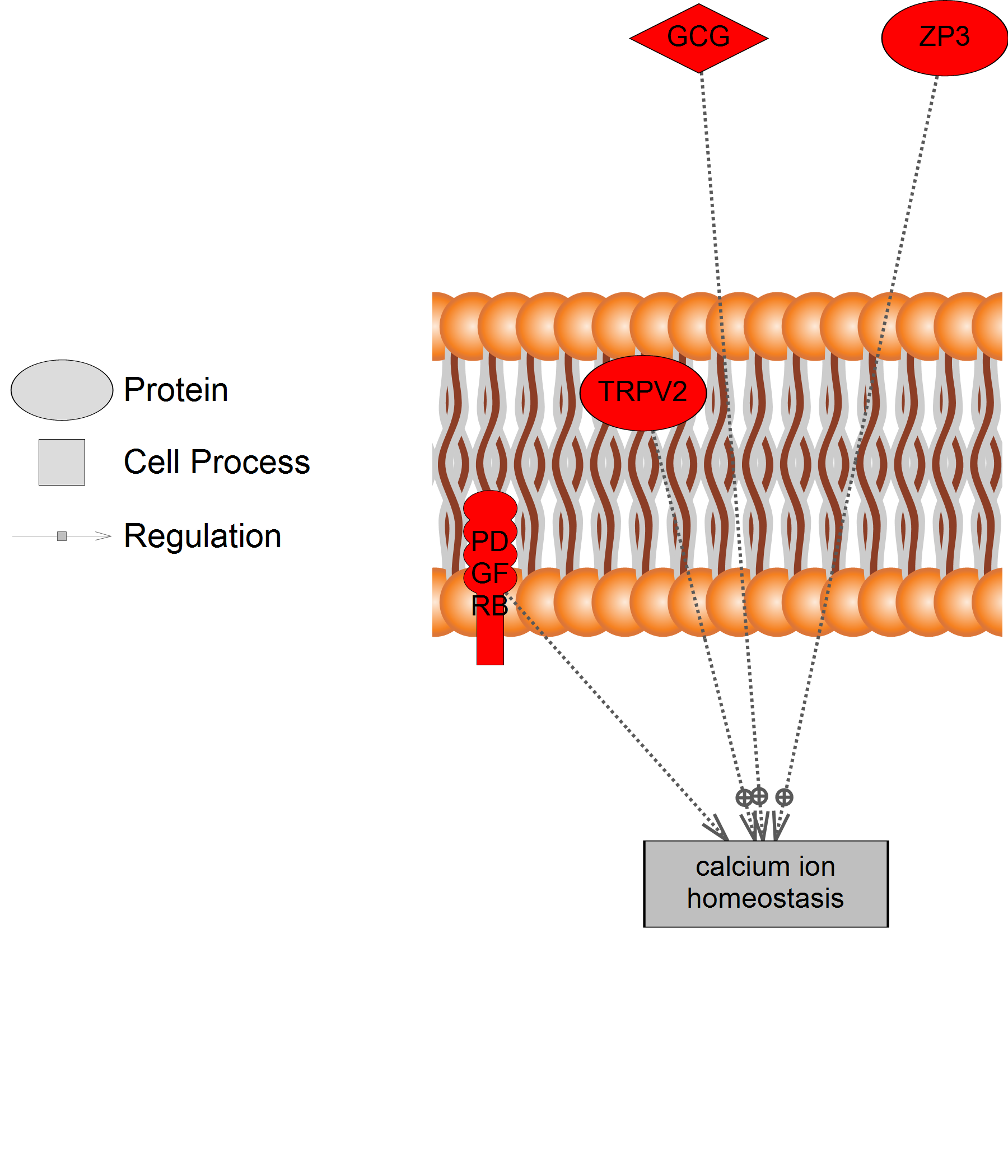

Supplement: Figure S4 — Factors involved in Ca2+ homeostasis; red indicates that the gene is increased in transcript level. (TIFF) [file pone.0104518.s004.tiff]
