# Supplementary material for: Transcriptional Profiling in Rat Hair Follicles following Simulated Blast Insult: A New Diagnostic Tool for Traumatic Brain Injury
Source: PLoS One. 2014 Aug 19;9(8):e104518. doi: 10.1371/journal.pone.0104518 (PMC4138085; doi:10.1371/journal.pone.0104518)
Supplement: Table S1 — Primers and their corresponding conditions for qPCR analysis. (DOCX) [file pone.0104518.s006.docx]

Table S1 Primers and their corresponding conditions for qPCR analysis

| **GenBank ID** | **Gene Name** | **Direction** | **Primer Sequence (5'to 3')** | **Annealing**  **Temp (°C)** | **Amplicon Size** | **Primer**  **Conc (nM)** |
| --- | --- | --- | --- | --- | --- | --- |
| NM_001012006 | Ace2 | fw | TGGACAACTTCTTGACAGCCC | 60 | 93 | 350 |
|  |  | rv | ACCCTTCATTGGCTCCGTTT | 60 | 93 | 350 |
| NM_198769 | Tlr2 | fw | TGAGAATGATGTGGGCGTGG | 58 | 94 | 350 |
|  |  | rv | TCGCTGTAGGAAACAAAGGCA | 58 | 94 | 350 |
| NM_001013231 | Pea15a | fw | CAGTGGGGAAGGAAGGTCAA | 58 | 91 | 350 |
|  |  | rv | CCCGTAACAAGGGCATAAAGG | 58 | 91 | 350 |
| NM_001191951 | Ahnak | fw | TCAGGTTCAAAGGGAGAGGGA | 60 | 94 | 350 |
|  |  | rv | CAGGCAGATTGACATCCCCA | 60 | 94 | 350 |
| NM_001126099 | Triap1 | fw | TGTGTCCAGAAAGCAATCAAGG | 58 | 101 | 350 |
|  |  | rv | CAGGCAGGTCAAGAGGAGTT | 58 | 101 | 350 |
| NM_001106615 | Arpc4 | fw | GAACGACACAACAAGCCAGAG | 58 | 109 | 350 |
|  |  | rv | CCTTTTCCTTCTCATTCCTGCT | 58 | 109 | 350 |
| NM_017064 | Stat5a | fw | CCCGTGTCAGTTGTATCCCT | 58 | 92 | 350 |
|  |  | rv | ACCACTACTTTAGCCAGACCC | 58 | 92 | 350 |
| NM_001009624 | Ska2 | fw | AGTCTCACGTGCCAGACGTA | 60 | 105 | 350 |
|  |  | rv | CAGCCCCCAACCTCTAGACA | 60 | 105 | 350 |
| NM_022519 | Serpina1 | fw | GCATCTGGAGCAAACTCTCAC | 58 | 101 | 350 |
|  |  | rv | CAGAGATGGACAGTTTGGGGAA | 58 | 101 | 350 |
| NM_033234 | Hbb | fw | GGGGAAAGGTGAACCCTGAT | 58 | 102 | 350 |
|  |  | rv | ACAGGTCCCCAAAGCTATCAA | 58 | 102 | 350 |
| BC072542.1 | Elf-alpha | fw | ATTCCACCGAGCCACCATAC | 58 | 180 | 350 |
|  |  | rv | CTTTCCATCCCTTGAACCAC | 58 | 180 | 350 |
