# Supplementary material for: Transcriptional Profiling in Rat Hair Follicles following Simulated Blast Insult: A New Diagnostic Tool for Traumatic Brain Injury
Source: PLoS One. 2014 Aug 19;9(8):e104518. doi: 10.1371/journal.pone.0104518 (PMC4138085; doi:10.1371/journal.pone.0104518)
Supplement: Table S2 — A: Enriched GO terms in rat hair follicles after blast exposure under the domain of Biological Processes. Table S2B: Enriched GO terms in rat hair follicles after blast exposure under the domain of Molecular Functions. Table S2C: Enriched GO terms in rat hair follicles after blast exposure under the domain of Cellular Components. (DOCX) [file pone.0104518.s007.docx]

Table S2A Enriched GO terms in rat hair follicles after blast exposure under the domain of Biological Processes

|  | | **Accession** | | **GO term** |
| --- | --- | --- | --- | --- |
| **Signaling transduction** | | GO:0007200 | | Activation of phospholipase c activity by g-protein coupled receptor protein signaling pathway coupled to ip3 second messenger |
|  | | GO:0007186 | | G-protein coupled receptor protein signaling pathway |
|  | | GO:0034220 | | Ion transmembrane transport |
|  | | GO:0006811 | | Ion transport |
|  | | GO:0002755 | | Myd88-dependent toll-like receptor signaling pathway |
|  | | GO:0043124 | | Negative regulation of I-kappab kinase-NF-kappab cascade |
|  | | GO:0043407 | | Negative regulation of map kinase activity |
|  | | GO:0018107 | | Peptidyl-threonine phosphorylation |
|  | | GO:0010535 | | Positive regulation of activation of jak2 kinase activity |
|  | | GO:0090280 | | Positive regulation of calcium ion import |
|  | | GO:0043268 | | Positive regulation of potassium ion transport |
|  | | GO:0032321 | | Positive regulation of rho gtpase activity |
|  | | GO:0045880 | | Positive regulation of smoothened signaling pathway |
|  | | GO:0043113 | | Receptor clustering |
|  | | GO:0051592 | | Response to calcium ion |
|  | | GO:0010038 | | Response to metal ion |
|  | | GO:0050896 | | Response to stimulus |
|  | | GO:0048384 | | Retinoic acid receptor signaling pathway |
|  | | GO:0051403 | | Stress-activated mapk cascade |
|  | | GO:0008063 | | Toll signaling pathway |
|  | | GO:0034130 | | Toll-like receptor 1 signaling pathway |
|  | | GO:0034134 | | Toll-like receptor 2 signaling pathway |
|  | | GO:0034138 | | Toll-like receptor 3 signaling pathway |
|  | | GO:0034142 | | Toll-like receptor 4 signaling pathway |
|  | | GO:0002224 | | Toll-like receptor signaling pathway |
|  | |  | |  |
| **Inflammatory responses** | | GO:0006954 | Inflammatory response | |
|  | | GO:0032700 | Negative regulation of interleukin-17 production | |
|  | | GO:0032729 | Positive regulation of interferon-gamma production | |
|  | | GO:0032757 | Positive regulation of interleukin-8 production | |
|  | | GO:0045084 | Positive regulation of interleukin-12 biosynthetic process | |
|  | | GO:0032819 | Positive regulation of natural killer cell proliferation | |
|  | |  |  | |
| **CNS responses** | GO:0050804 | | | Regulation of synaptic transmission |
|  | \| GO:0007268 \| \| --- \| | | | Synaptic transmission |
|  |  | | |  |
| **Cell survival/Proliferation** | GO:0045767 | | | Anti-apoptosis Positive regulation of glutamate secretion |
|  | GO:0001906 | | | Cell killing |
|  | GO:0006968 | | | Cellular defense response |
|  | \| GO:0006952 \| \| --- \| | | | Defense response |
|  | GO:0042693 | | | Muscle cell fate commitment |
|  | GO:0043154 | | | Negative regulation of caspase activity |
|  | GO:0033033 | | | Negative regulation of myeloid cell apoptosis |
|  | GO:0043069 | | | Negative regulation of programmed cell death |
|  | GO:0048662 | | | Negative regulation of smooth muscle cell proliferation |
|  | GO:0043065 | | | Positive regulation of apoptosis |
|  | GO:0042026 | | | Protein refolding |
|  | \| GO:0051726 \| \| --- \| | | | Regulation of cell cycle |
|  | GO:0009612 | | | Response to mechanical stimulus |
|  |  | | |  |
| **Other TBI responses** | GO:0006953 | | | Acute-phase response |
|  | GO:0019370 | | | Leukotriene biosynthetic process |
|  | GO:0006402 | | | mRNA catabolic process |
|  | GO:0010466 | | | Negative regulation of peptidase activity |
|  | GO:0014049 | | | Positive regulation of glutamate secretion |
|  | GO:0034105 | | | Positive regulation of tissue remodelling |
|  | GO:0050880 | | | Regulation of blood vessel size |
|  | GO:0040012 | | | Regulation of locomotion |
|  | GO:0000302 | | | Response to reactive oxygen species |

Table S2B Enriched GO terms in rat hair follicles after blast exposure under the domain of Molecular Functions

|  | **Accession** | **Go term** |
| --- | --- | --- |
| **Signaling transduction** | GO:0015662 | Atpase activity, coupled to transmembrane movement of ions, phosphorylative mechanism |
|  | GO:0008603 | Camp-dependent protein kinase regulator activity |
|  | GO:0043499 | Eukaryotic cell surface binding |
|  | GO:0004930 | G-protein coupled receptor activity |
|  | GO:0015280 | Ligand-gated sodium channel activity |
|  | GO:0017046 | Peptide hormone binding |
|  | GO:0019870 | Potassium channel inhibitor activity |
|  | GO:0003700 | Sequence-specific DNA binding transcription factor activity |
|  | GO:0004871 | Signal transducer activity |
|  |  |  |
| **Inflammatory responses** | GO:0005125 | Cytokine activity |
|  | GO:0005126 | Cytokine receptor binding |
|  |  |  |
| **Other TBI responses** | GO:0004869 | Cysteine-type endopeptidase inhibitor activity |
|  | GO:0016712 | Flavoprotein monooxygenase activity |
|  | GO:0016594 | Glycine binding |
|  | GO:0004051 | Lipoxygenase activity |
|  | GO:0005537 | Mannose binding |
|  | GO:0004497 | Monooxygenase activity |
|  | GO:0004984 | Olfactory receptor activity |
|  | GO:0016491 | Oxidoreductase activity |
|  | GO:0008233 | Peptidase activity |
|  | GO:0030414 | Peptidase inhibitor activity |
|  | GO:0002020 | Protease binding |
|  | GO:0001972 | Retinoic acid binding |
|  | GO:0004867 | Serine-type endopeptidase inhibitor activity |

Table S2C Enriched GO terms in rat hair follicles after blast exposure under the domain of Cellular Components

| **Accession** | **GO term** |
| --- | --- |
| GO:0045178 | Basal part of cell |
| GO:0032059 | Bleb |
| GO:0043218 | Compact myelin |
| GO:0005788 | Endoplasmic reticulum lumen |
| GO:0031012 | Extracellular matrix |
| GO:0005576 | Extracellular region |
| GO:0005615 | Extracellular space |
| GO:0005577 | Fibrinogen complex |
| GO:0005833 | Hemoglobin complex |
| GO:0046581 | Intercellular canaliculus |
| GO:0031430 | M band |
| GO:0000228 | Nuclear chromosome |
| GO:0005886 | Plasma membrane |
| GO:0005886 | Platelet alpha granule membrane |
| GO:0005578 | Proteinaceous extracellular matrix |
| GO:0016529 | Sarcoplasmic reticulum |
| GO:0030141 | Stored secretory granule |
